# Comparative study of posaconazole and voriconazole for primary antifungal prophylaxis in patients with pediatric acute leukemia

**DOI:** 10.1038/s41598-023-46328-0

**Published:** 2023-11-01

**Authors:** Songji Tu, Kunlong Zhang, Ningling Wang, Jinhua Chu, Linhai Yang, Zhiwei Xie

**Affiliations:** Department of Pediatrics, The Second Affiliated Hospital of Anhui Medical University, 678 Furong Road, Economic and Technological Development Zone, Shushan District, Hefei, 230601 China

**Keywords:** Medical research, Oncology

## Abstract

Posaconazole and voriconazole are commonly used for preventing invasive fungal disease (IFD), but few studies compared posaconazole with voriconazole for primary antifungal prophylaxis (PAP) in pediatric acute leukemia. To compare posaconazole with voriconazole for PAP in pediatric acute leukemia. This retrospective observational study enrolled pediatric patients with non-M3 acute myeloid leukemia (AML) or acute lymphoblastic leukemia (ALL) between December 2017 and November 2019 in the Second Affiliated Hospital of Anhui Medical University. The patients received voriconazole or posaconazole for PAP. The primary outcome was the breakthrough of IFD. The secondary outcome was the overall survival (OS) and IFD-free survival of patients. A total of the 275 patients were enrolled, of which 120 patients taking voriconazole (43.6%) and 155 patients taking posaconazole (56.4%). The breakthrough of IFD occurred in 19 (15.8%) patients taking voriconazole and in 12 (7.7%) patients taking posaconazole (*P* = 0.035). There was no significant differences in IFD-free survival (*P* = 0.336) or OS (*P* = 0.069) between the patients taking voriconazole and posaconazole. In the subgroup of AML patients, the OS of patients taking posaconazole was better than those receiving voriconazole (*P* = 0.017). Posaconazole and voriconazole were comparable for PAP in patients with pediatric acute leukemia regarding the OS and IFD-free survival, but posaconazole might achieve a lower IFD breakthrough rate.

Invasive fungal disease (IFD) can be a major complication of acute leukemia treatment, and is associated with significant morbidity and mortality^1,2^. The most common pathogens of IFD in childhood leukemia are *Aspergillus* and *Candida*^3,4^. The annual incidence of invasive candidiasis in children is 0.92 per 100,000^5^, but the risk increases in children with acute leukemia^6,7^. Increased intensity of therapy, such as chemotherapy and organ transplantation, have coincided with a substantial increase in the incidence of invasive fungal infections^8^.

Systemic antifungal prophylaxis can be an effective approach to reducing IFD, and primary antifungal prophylaxis (PAP) was recommended by the guidelines^9^. Posaconazole is approved for the prophylaxis of *Aspergillus* and *Candida* infections. In addition, posaconazole is used to treat oropharyngeal candidiasis, typically for patients refractory to treatment with fluconazole and itraconazole^10–12^. Voriconazole is approved for invasive aspergillosis, candidemia in non-neutropenic patients, esophageal candidiasis, and disseminated candidiasis^10–12^.

A systematic review and network meta-analysis showed that voriconazole might be the best choice for patients undergoing HSCT, and posaconazole might be the best prophylactic option for patients with AML or MDS^13^. Another study showed that posaconazole and voriconazole are effective in preventing IFDs in adult patients with hematological malignancy, but symptomatic adverse events were more common with voriconazole^14^. A cost-effectiveness analysis suggested a better cost-effectiveness of posaconazole compared to voriconazole for IFD prophylaxis in AML^15^. Overall, posaconazole and voriconazole are recommended as the most reasonable options for the prevention of IFD. However, there were few studies on posaconazole and voriconazole for PAP in pediatric acute leukemia.

Therefore, this study aimed to compare the efficacy of using posaconazole and voriconazole for PAP in pediatric acute leukemia.

## Methods

### Study design and participants

This retrospective, observational study included pediatric patients with non-M3 AML and acute lymphoblastic leukemia (ALL) , treated with PAP between December 2017 and November 2019 in the Department of Pediatric Hematology and Oncology of the Second Affiliated Hospital of Anhui Medical University (Hefei, China).

The inclusion criteria were (1) patients diagnosed with non-M3 AML or ALL with ≤ 14 years of age, (2) underwent chemotherapy, including induction therapy and consolidation therapy regimens, (3) received oral voriconazole or posaconazole for PAP, and (4) None received any antibacterial prophylaxis. The exclusion criteria were (1) received other drugs or intravenous preparations of voriconazole or posaconazole for PAP or (2) with a previous history of invasive fungal infection.

### Treatment details

All children received only chemotherapy for acute leukemia. And they received voriconazole or posaconazole orally for PAP, which was started when neutropenia occurred (defined as neutrophil counts < 0.5 × 10^9^/L). Prophylaxis dosing method of the voriconazole group: weight < 50 kg, 9 mg/kg/dose every 12 h, single dose not to exceed 200 mg; weight ≥ 50 kg, 200 mg/dose every 12 h, and of the posaconazole group: 4 mg/kg/dose, three times a day, with the highest dose not exceeding 200 mg/dose, three times a day. The PAP was administered until the neutrophil count increased to ≥ 0.5 × 10^9^/L or a breakthrough IFD occurred. Medical notes were reviewed by the chief physician and attending physician. Drug concentration monitoring was not performed during administration. All treatments were provided on conventional wards without laminar flow.

Basic demographic and clinical characteristics were collected, including age, sex, subtype of leukemia, PAP strategy, the time of IFD breakthrough, and infection site.

### Outcomes

The primary outcome was breakthrough of IFD (included proven IFD and probable IFD only). The secondary outcome was the patient prognosis, including IFD-free survival and overall survival (OS).

The breakthrough of IFD was diagnosed according to the definition of the consensus group of the European Organization for Research and Treatment of Cancer Invasive Fungal Infections Cooperative Group (EORTC)^16^ between at least 7 days after the start of PAP and 7 days after the end of PAP. IFD-free survival was defined as the time between treatment initiation and breakthrough of IFD. The OS was defined as the time between treatment initiation and death from any cause. The follow-up period ranged from the start of treatment to the last follow-up time.

### Statistical analysis

Data were analyzed using SPSS 22.0 (IBM Corp., Armonk, NY, USA). Quantitative variables were presented as medians (ranges) and analyzed using the Mann–Whitney U-test. Categorical variables were presented as percentages (%) and analyzed using the chi-square test or Fisher's exact test. IFD-free survival and OS were determined using the Kaplan–Meier method, and the log-rank test was used to compare the curves. A two-sided P < 0.05 was considered statistical significant.

## Results

This study included 275 pediatric patients with acute leukemia and aged 1 to 14 years. Among them, 219 (79.6%) diagnosed with ALL, and 56(20.4%) with non-M3 AML. There were 155 patients taking posaconazole (56.4%), and 120 patients taking voriconazole (43.6%). Age, sex, and diagnosis were comparable between the patients taking voriconazole and posaconazole (all *P* > 0.05) (Table 1).

**Table 1:** Demographic and clinical characteristics of the patients.

Characteristics	Voriconazole (n = 120)	Posaconazole (n = 155)	P
Age [years, median (range)]	6 (1–14)	6 (1–14)	0.116
Sex, n (%)			0.281
Male	65 (54.2)	94 (60.6)	
Female	55 (45.8)	61 (39.4)	
Diagnosis, n (%)			0.462
Acute lymphoblastic leukemia	98 (81.7)	121 (78.1)	
Acute myeloid leukemia	22 (18.3)	34 (21.9)	
Neutropenia duration [days, median (range)]	12 (5–36)	14 (7–51)	0.161
Prolonged use of corticosteroid			0.927
Absence	30(25.0)	38(24.5)	
Presence	90(75.0)	117(75.5)	

**Figure 1:**
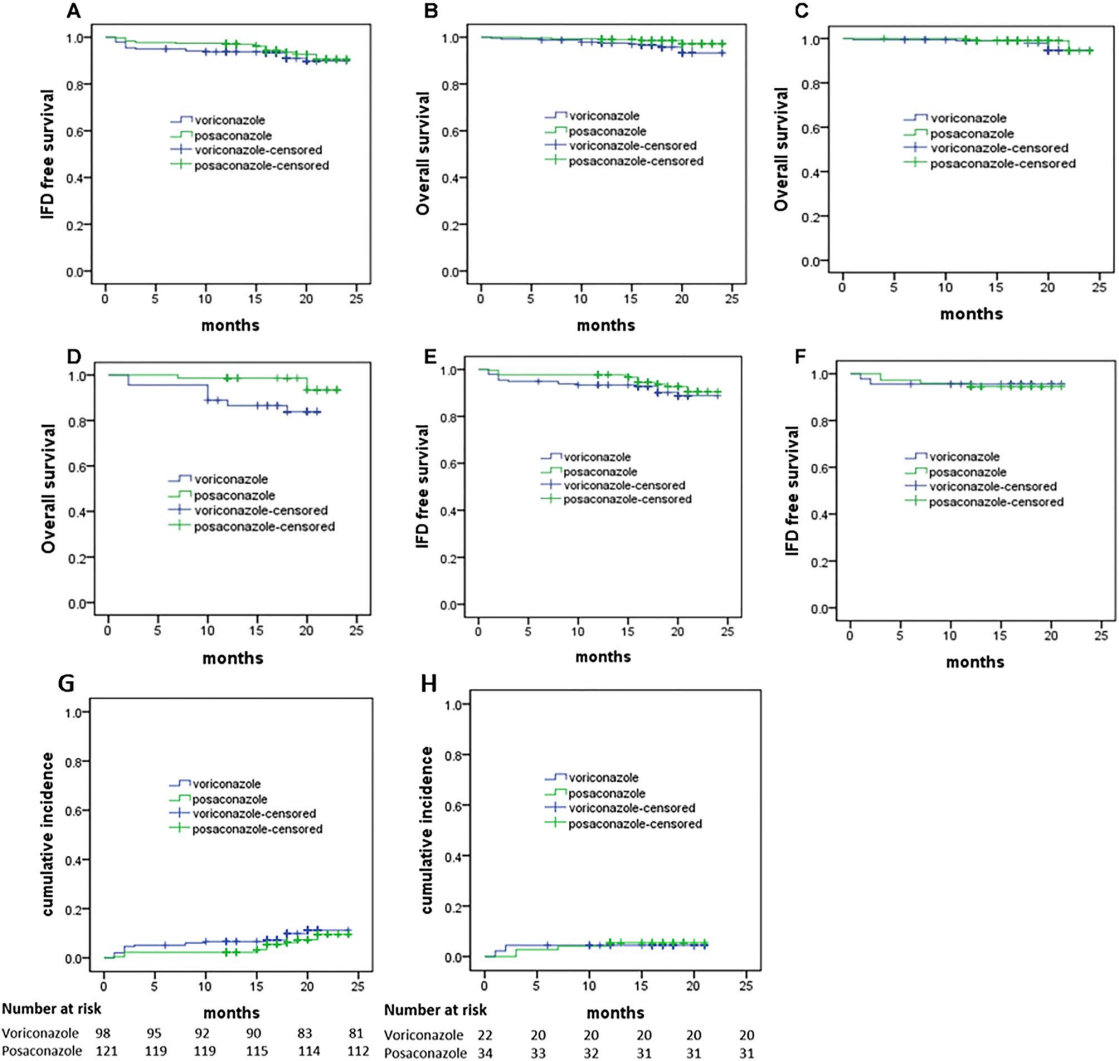
Kaplan–Meier plots for invasive fungal disease (IFD)-free survival and overall survival (OS). (**A**) IFD-free survival in all patients (*P* = 0.336). (**B**) OS in all patients (*P* = 0.069). (**C**) OS in acute lymphoblastic leukemia (ALL) (*P* = 0.251). (**D**) OS in acute myeloid leukemia (AML) (*P* = 0.017). (**E**) IFD-free survival in ALL (*P* = 0.267). (**F**) IFD-free survival in AML (*P* = 0.851). (**G**) Cumulative incidence of IFD in ALL. (**H**) Cumulative incidence of IFD in AML.

Breakthrough of IFD occurred in 19 (15.8%) patients receiving voriconazole and in 12 (7.7%) patients receiving posaconazole (*P* = 0.035). There were 16 patients that developed IFD breakthrough during the induction stage, and 11 (9.2%) of them were treated with voriconazole, the other 5 (3.2%) were treated with posaconazole (*P* = 0.473). Most IFDs were in the lungs, with 17 (14.2%) in patients receiving voriconazole, and 8 (5.2%) in patients receiving posaconazole (*P* = 0.147) (Table 2). There were no significant differences in IFD-free survival (*P* = 0.336) or OS (*P* = 0.069) between the patients receiving voriconazole and posaconazole (Fig. 1A,B).

**Table 2:** Patient outcomes.

Outcomes	Voriconazole (n = 120)	Posaconazole (n = 155)	*P*
Breakthrough of IFD, n (%)	19 (15.8)	12 (7.7)	0.035
Time of IFD breakthrough, n (%)			0.473
Induction stage	11 (9.2)	5 (3.2)	
Other	8 (6.7)	7 (4.5)	
Infection site, n (%)			0.147
Lung	17 (14.2)	8 (5.2)	
Other	2 (1.7)	4 (2.6)

In the subgroup of AML patients, the OS of patients taking posaconazole was better than those taking voriconazole (*P* = 0.017), but there were no significant differences in the IFD-free survival (*P* = 0.851) (Fig. 1D,F). In the subgroup of ALL patients, the IFD-free survival (*P* = 0.267) and OS (*P* = 0.251) were not significantly different between the patients taking posaconazole or voriconazole (Fig. 1E,C).

## Discussion

This study compared voriconazole with posaconazole for PAP in pediatric acute leukemia. The rate of breakthrough IFD during PAP was lower in the posaconazole group compared with (7.7% vs. 15.8%), which was higher than that in a previous study of adult patients with AML, ALL, and MDS (2.5% vs. 4.8%)^17^. Hachem et al.^14^ observed breakthrough IFDs in 3% and 0% of acute leukemia patients treated with posaconazole and voriconazole. It might be related to the environment in which the children were treated, and the definition of a breakout IFD may also be an influencing factor. This study used a standard definition of breakthrough IFD, which occurring between at least 7 days after the start of PAP and 7 days after the end of PAP^16^. In addition, children might be more susceptible to IFDs than adults because of a more immature immune system^18,19^.

The results suggested that the IFD-free survival or OS was not significantly different between the two treatments, but posaconazole might achieve a lower IFD breakthrough rate for PAP in pediatric patients with acute leukemia than voriconazole. The results might help the selection of drugs for the prophylactic management of IFDs in patients with pediatric acute leukemia.

Remission induction chemotherapy has been suggested to be the highest-risk phase for the development of IFD in patients undergoing initial treatments^20,21^. Several studies suggested that most IFDs in ALL and AML occurred during the induction stage and with stronger chemotherapy^22–24^. Similarly, in this study, 16 cases (16/31, 51.6%) of breakthrough IFDs occurred during induction. It can be related to severe neutropenia and high dosages of steroids^25^.

Some studies showed that proven and probable invasive aspergillosis is the most common infection in AML patients receiving active triazole PAP after intensive chemotherapy^26–28^. For patients with suspected IFD, computed tomography (CT) of the lungs and other investigations are recommended^29^. CT plays an important role in diagnosing and managing patients with fungal infections due to its ability to reveal early predictive signs of fungal infection^30^. This study showed that in 25 cases (25/31, 80.6%), the location of breakthrough IFD was in the lungs.

Patients with IFD often have to interrupt, delay, or change the chemotherapy regimens, which can undermine their long-term survival^31^. Kobayashi et al.^32^ found significantly lower survival in patients with IFD than those without IFD in children and adolescents with hematological malignancies, other malignant diseases, and aplastic anemia. Still, the effect of PAP on OS is unclear. Dahlen et al.^26^ found that posaconazole prophylaxis decreased the incidence of IFD but did not improve short-term OS. Another study concluded that PAP could reduce long-term mortality in salvaged patients needing successive treatment, such as allogeneic HSCT^33^. In this study, the choice of either posaconazole or voriconazole for primary prophylaxis had no significant effect on OS or IFD-free survival, which might related to the fact that the number of patients experiencing endpoint events was limited and the median survival time had not been reached. On the other hand, in the subgroup analysis of AML patients, the OS of patients in posaconazole group was better than those in voriconazole group. These results are supported by a cost-effectiveness analysis suggested a better cost-effectiveness of posaconazole vs. voriconazole for IFD prophylaxis in patients with AML, including a lower death rate, suggesting better patient outcomes with posaconazole and a smaller use of healthcare resources^15^.

This study had several limitations. First, it was a single-center retrospective study, and the number of patients was relatively small. The data that could be analyzed were limited to those in the patient charts. Second, microbiological identification of patients with breakthrough, as well as the plasma concentrations of posaconazole and voriconazole were not tested. Hence, the possibility of low concentrations in children with breakthrough IFD could not be confirmed. Third, the median survival time had not been reached, which might because the follow-up period was not long enough, and long-term follow-up was needed in future studies.

In conclusion, posaconazole and voriconazole were comparable for PAP in patients with pediatric acute leukemia regarding the OS and IFD-free survival, but posaconazole might achieve a lower IFD breakthrough rate. Multicenter clinical trial with large samples were needed in the future to confirm the results.

## Ethical approval

This work has been carried out in accordance with the Declaration of Helsinki (2000) of the World Medical Association. This study was approved by the Ethics Committee of the Second Affiliated Hospital of Anhui Medical University (PJ-YX201501). This article is a retrospective study. Therefore, the Institutional waived the requirement to obtain distinct written informed consent from the patients.

## Data Availability

The datasets used and/or analyzed during the current study are available from the corresponding author upon reasonable request.
